# Addressing the UHC Challenge Using the Disease Control Priorities 3 Approach: Lessons Learned and an Overview of the Pakistan Experience

**DOI:** 10.34172/ijhpm.2023.8003

**Published:** 2023-12-16

**Authors:** Ala Alwan, Sameen Siddiqi, Malik Safi, Raza Zaidi, Muhammad Khalid, Rob Baltussen, Ina Gudumac, Maryam Huda, Maarten Jansen, Wajeeha Raza, Sergio Torres-Rueda, Wahaj Zulfiqar, Anna Vassall

**Affiliations:** ^1^DCP3 Country Translation Project, London School of Hygiene and Tropical Medicine, London, UK; ^2^Department of Community Health Sciences, Aga Khan University, Karachi, Pakistan; ^3^Ministry of National Health Services, Regulations and Coordination, Islamabad, Pakistan; ^4^Department of Health Evidence, Radboud Institute of Health Sciences, Radboud University Medical Center, Nijmegen, The Netherlands; ^5^Centre for Health Economics, University of York, York, UK; ^6^Department of Global Health & Development, London School of Hygiene and Tropical Medicine, London, UK

**Keywords:** Essential Package of Health Services, Health Benefit Package, Universal Health Coverage, Disease Control Priorities 3, Pakistan

## Abstract

**Background:** Pakistan developed its first national Essential Package of Health Services (EPHS) as a key step towards accelerating progress in achieving Universal Health Coverage (UHC). We describe the rationale, aims, the systematic approach followed to EPHS development, methods adopted, outcomes of the process, challenges encountered, and lessons learned.

**Methods:** EPHS design was led by the Ministry of National Health Services, Regulations & Coordination. The methods adopted were technically guided by the Disease Control Priorities 3 Country Translation project and existing country experience. It followed a participatory and evidence-informed prioritisation and decision-making processes.

**Results:** The full EPHS covers 117 interventions delivered at the community, health centre and first-level hospital platforms at a per capita cost of US$29.7. The EPHS also includes an additional set of 12 population-based interventions at US$0.78 per capita. An immediate implementation package (IIP) of 88 district-level interventions costing US$12.98 per capita will be implemented initially together with the population-based interventions until government health allocations increase to the level required to implement the full EPHS. Interventions delivered at the tertiary care platform were also prioritised and costed at US$6.5 per capita, but they were not included in the district-level package. The national EPHS guided the development of provincial packages using the same evidence-informed process. The government and development partners are in the process of initiating a phased approach to implement the IIP.

**Conclusion:** Key ingredients for a successful EPHS design requires a focus on package feasibility and affordability, national ownership and leadership, and solid engagement of national stakeholders and development partners. Major challenges to the transition to implementation are to continue strengthening the national technical capacity, institutionalise priority setting and package design and its revision in ministries of health, address health system gaps and bridge the current gap in financing with the progressive increase in coverage towards 2030.

## Background

**Key Messages**

**Implications for policy makers**

- Key ingredients for a successful transition from package design to rollout are national ownership and leadership, stakeholder engagement, affordability, feasibility of implementation along the universal health coverage (UHC) timeline, and institutionalisation of the process.
- Evidence informed decision-making in priority-setting of interventions and package design processes can be successfully achieved in low- and lower-middle income countries (LLMICs) despite scarcity of local data and limited technical capacity in evidence collection, analysis, and use. The Pakistan experience builds on the global Disease Control Priorities 3 (DCP3) evidence and offers a methodological approach to design national essential package of health services (EPHS). However, every country will need to delineate its own path to developing the EPHS.
- Important lessons learned for future consideration are a more robust process of community involvement in decision-making, a stronger engagement of the planning and finance sectors, and in-depth assessment of existing healthcare delivery systems conducted concurrently with package design. Capacity building, including learning-by-doing, can be further strengthened. Institutionalisation of existing skills is a challenge in the longer-term.

**Implications for the public**

The Pakistan experience shows that priority setting and package design have been at the centre of the national initiative on universal health coverage (UHC). The role and active engagement of the community and civil society organisations in dialogue and decision-making processes is a critical component that should have been more prominent during the package design process. Many countries have developed packages of essential health services, yet they have not been implemented. This paper identifies factors that contribute to a successful package design and transition to implementation. By investing in publicly funded high priority interventions, access to essential care will be improved and financial risks to the population can be reduced. However, unrealistically costly packages beyond what the government can afford is one of the reasons that impede roll-out. UHC packages must also be consistent with population healthcare needs and health system realities.

At least half of the world’s population still lacks access to essential health services, while about 100 million people are being impoverished by health expenses every year.^1,2^ Almost a billion or more than 13% of the world’s population spends more than 10% of their household income on health.^2^ As part of the Sustainable Development Goals (SDGs), United Nations (UN) Member States agreed in 2015 to achieve universal health coverage (UHC) by 2030.^3^ This commitment was reaffirmed in 2019 during a special high-level meeting of the UN General Assembly on UHC,^4^ when Heads of State and Government pledged to scale up efforts to improve access to essential health services. A key step in the roadmap to UHC is for countries to develop an essential package of health services (EPHS) that is evidence informed, feasible, of high impact, and accessible to all.

 The Disease Control Priorities (DCP) initiative, published by the World Bank, has endeavoured over the last three decades to assist countries in assessing evidence and value for money of health interventions to support decision-making on resource allocations.^5-7^ The first effort was launched in the 1993 as the World Development Report^5^ and the second edition was released in 2006^6^. A decade later, the third edition (DCP3), launched in 2017, provides an up-to-date review of priority health interventions through a systematic appraisal of evidence, new economic analyses, and expert judgment across 21 health areas, with the goal of influencing resource allocation at country level.^7^

 The DCP3 concept focuses on meeting the three UHC dimensions of expanding population coverage, improving access to a broader range of essential services, and reducing financial risk. In this respect, the challenge is to determine which services should be considered highest priority in the context of limited resources.

 What makes DCP3 unique is the focus on UHC and the emphasis on supporting low- and lower-middle income countries (LLMICs) in developing essential health packages. The criteria adopted for selecting interventions during package design include evidence of impact, cost-effectiveness, financial risk protection, equity, and feasibility for implementation in LLMICs. The aim is to serve as a reference for formulating country-specific UHC packages. DCP3 proposes two generic packages: an essential UHC package (EUHC) of 218 interventions for lower middle-income countries and a highest-priority package of 108 interventions to serve the immediate needs of low-income countries, where the fiscal space for health is more constrained and too limited to cover the entire EUHC package.^8,9^ In 2018, a DCP3 Country Translation project was established at the London School of Hygiene and Tropical Medicine to support pilot countries in using the DCP3 evidence and packages to guide the development of their own UHC packages and to build their technical capacity in priority setting and package design. The project also aims to update and reinforce global guidance for other LLMICs in the development and implementation of UHC EPHS.

 Pakistan met the criteria set by the DCP3 Country Translation Project for selecting countries. These include a clear political commitment to UHC, engagement of relevant health and non-health sectors (specially finance and planning), and presence of an active unit with a mandate in priority setting and economic evaluation within and outside of the Ministry of Health.

 Pakistan, a lower middle-income country, has an estimated population of 221 million^10^ and a decentralised system with the governance of health largely devolved to provinces.^11^ Despite increases in government allocation to health, per capita health spending in 2017/2018 was US$ 39, which was primarily financed domestically (over 99%) and through out-of-pocket expenditure (53.8%).^12^ Public sector expenditure on health accounted for 35% of the overall current health spending. In 2018, Pakistan initiated a project to develop a national EPHS, drawing on the DCP3 evidence and approach.

 The aim of this paper is to provide an outline of the objectives of the project, the methods adopted, and the policy processes followed in this experience. It offers a methodological approach to designing national EPHS and presents the final UHC packages of essential health services, together with the challenges encountered and the lessons learned. The paper is supported by four companion papers that detail the appraisal, assessment, costing and decision-making processes followed to develop the Pakistan EPHS.^13-16^

## Methods

###  Principles and Methodological Approach 

 The process started in 2019 and was led by the Ministry of National Health Services, Regulations & Coordination (MNHSR&C), with technical guidance and support from the DCP3 Country Translation Project. The design of the package was guided by a set of key principles that included transparency, national ownership and execution, focus on feasibility and affordability, and inclusivity by engaging public sector institutions, non-governmental stakeholders, and development partners. The process spanned over two years, starting with the decision of the Inter-Ministerial Population & Health Council in September 2018 to use the DCP3 evidence in developing its first UHC package. Most of the work to design the package was conducted between 2019 and 2020. This period also included intensive capacity building in priority setting and package design both in the MNHSR&C and in partner institutions.

 The formal process for developing the EPHS covered a series of consultations with stakeholders leading to agreement on the objectives, expected outcomes, and methods of work. A roadmap was developed to guide the priority setting and package design processes. Box 1 summarises the main steps adopted in developing the Pakistan EPHS. The first eight steps have been rigorously followed in developing the package, step 9 is currently being piloted in one province, and step 10 is envisaged as more experience is gained following EPHS implementation.

**Box 1. Ten Steps for Setting the Essential Package of Health Services in Pakistan**

- Assessment of the disease burden, health service needs, health system priorities, and current financing landscape.
- Establishing a governance structure for dialogue, evidence-informed deliberation on priorities and services, and decision-making.
- Reaching consensus on decision criteria and collect evidence for setting priorities and selecting health interventions.
- Implementing an evidence informed decision-making process to prioritise health interventions and decide what to include and what to exclude.
- Conducting detailed costing of the prioritised interventions based on current and target coverage levels, including the UHC target in 2030.
- Assessing the health system capacity to implement the package, especially at the district level, and identify actions to fill existing gaps and facilitate implementation.
- Assessing the budget impact of the package and link the EPHS to the budgeting and resource allocation process.
- Establishing a monitoring and evaluation framework to assess performance and outcomes.
- Adapting and piloting the package at the subnational level in devolved settings.
- Reviewing the package periodically based on policy change, new evidence, health system capacity, and available fiscal space.

--------------------

Abbreviations: EPHS, essential package of health services; UHC, universal health coverage.

Further description and information on the background and methodology, including data collection and analysis, criteria for prioritisation, appraisal of evidence, and analysis of costs are provided in the companion papers^13-16^ and the General Appendix, which is published as part of this collection of papers.

 The challenges encountered and lessons learned during the development of the Pakistan federal and provincial packages are drawn from the discussions made during National Advisory Committee (NAC) and International Advisory Group (IAG) meetings and the national technical meeting and satellite symposium organized in January 2023 in Islamabad. The January meeting discussed the experience gained, the challenges encountered, and the key steps for the transition to implementation and package roll-out. The conclusions presented in this paper are also informed by the DCP3 review of country experience in priority setting and UHC-related policies, which was conducted in 2021-2022 in six countries, including Pakistan. The country experience was reviewed by a network of 60 experts and professionals engaged in DCP3-related work and was published in a collection of papers in the British Medical Journal Global Health in 2023.^17-24^

###  Leadership and Coordination

 A governance structure (See General Appendix, Figure S1) was put in place by instituting a UHC EPHS Secretariat within the MNHSR&C. Key national partners included the Department of Community Health Sciences at the Aga Khan University and the Health Services Academy. Other key partners were the World Health Organization (WHO) and the Radboud University Medical Centre.

 The decision-making forums included (1) four technical working groups (TWGs) on reproductive, maternal, neonatal, child and adolescent health (RMNCAH), communicable diseases, non-communicable diseases (NCDs) and health services access, each with membership representing the different public health, health system and clinical professions; (2) the NAC, chaired by the Director General of Health; (3) the UHC EPHS Steering Committee (UHC-EPHS SC), chaired by the federal Minister of Health; (4) the Inter-ministerial Health and Population Council, which included the four provincial and two regional ministers of health as members; and (5) an IAG, comprised of international experts and DCP3 authors, with a mandate to review the process, methodologies, and contents of the EPHS.

###  Data Sources 

 The DCP3 EUHC list of 218 interventions served as a basis for setting up Pakistan’s package.^9^ Data required to inform the evidence sheets for prioritization of health services were collated from local, regional, and global secondary sources. Evidence on *burden of disease *was obtained from the Institute for Health Metrics and Evaluation.^25^ Evidence on *cost-effectiveness* was primarily derived from the Tufts Medical School Global Health Cost-Effectiveness Analysis registry,^26^ which compiles incremental cost-effectiveness ratio data on a large number of interventions. The remaining incremental cost-effectiveness ratios came from DCP3.

## Results

###  Mapping Existing Health Services and Current Coverage of Interventions

 A preliminary mapping of existing interventions in Pakistan’s public health sector was conducted against the DCP3 EUHC package of 218 interventions. The mapping revealed that only 135 (or 62%) of the 218 interventions were being implemented in public sector facilities (notwithstanding service quality) (See Supplementary file 1, Table S3). Among these, 42 interventions (31%) were generally available and 93 (69%) were being offered on a limited scale. The mapping clearly demonstrated major gaps in access to essential services in four clusters, including RMNCAH and communicable diseases and a lack of response to the epidemiological transition taking place in Pakistan. Of the generally available interventions, over three fourths were related to RMNCAH and communicable diseases and only 10% of the EUHC recommended interventions were implemented in the areas related to NCD, injury and health services access.

 An initial shortlist of 170 EUHC interventions was recommended for formal assessment and prioritisation.

###  Prioritising Interventions

 An important part of the prioritisation process was to define decision support criteria and gather evidence necessary for decision-making. Concurrent with the mapping exercise, a survey was conducted to identify criteria that would guide the prioritisation of essential interventions. Based on the survey results, consensus was reached on the following criteria: avoidable burden of disease, cost-effectiveness, financial risk protection, budget impact, equity, feasibility, and socio-economic impact. A more detailed information on the decision-making criteria is available in Baltussen et al^13^ and Box S1 in General Appendix.

 In assessing the criteria adopted, three were assessed using quantitative evidence: burden of disease, budget impact, and cost-effectiveness. Effectiveness was not seen as an important criterion because EUHC interventions were deemed effective *ex ante.* Other criteria were considered but data on these were insufficient to include in the quantitative assessment. Applicability of global cost-effectiveness evidence to the country context was systematically assessed using general and specific knock-out criteria. Huda et al describe the process used for adapting global estimates to the Pakistan context.^15^

 Regarding intervention costing, several costing approaches were debated. Primary data collection was considered but not preferred, as many DCP3 EUHC interventions were either not being delivered or were delivered at insufficient levels of quality. Similarly, databases such as those contained in DCP3 did not provide sufficient details on the sources and composition of costs presented and their appropriateness to the local context could not be validated. The team opted to develop a context-specific, normative, ingredients-based rapid method to estimate the unit costs of interventions. A bottom-up approach to costing was applied to community, health centre and the two hospital platforms, while a top-down approach was used for population-based interventions. The cost of each intervention and the package includes the health system resources directly used in service provision, but it did not include any indirect costs, above-service delivery, or other overheads. Similarly, the cost of governance at the district level was not included. The principles set out in the Global Health Costing Consortium reference case^27^ were followed. Raza et al^14^ provide further details on costing.

 For each of the 170 shortlisted DCP3 EUHC interventions, we reported the evidence on decision criteria to the TWGs and the NAC using a combination of intervention descriptions^28^ (Supplementary file 2, Figure S2) and evidence sheets (Supplementary file 2, Figure S3). The intervention descriptions sheets contained details on the delivery platform, process, providers, medicines, supplies, equipment, health information tools, supervision, availability of in-service training curriculum, and reference documents. The evidence sheets included information on burden of disease, cost-effectiveness and rank order, quality of cost-effectiveness evidence and budget impact for each intervention. We also presented the total costs, disability-adjusted life years (DALYs) averted, and a bookshelf of interventions, using a combination of the HIPTool^29^ and bespoke analyses.

###  Stages of Prioritisation

 Prioritisation was done in two stages. In the *first stage*, meetings were held by the TWGs (in November 2019 and February 2020) to prioritise and recommend interventions based on the agreed-upon decision criteria, without strict consideration for the available fiscal space for health. The interventions were initially prioritised and costed for community, health centre, first-level hospital, tertiary/referral hospital, and population-level platforms. This initial prioritisation resulted in the selection of a total of 151 interventions (26 at community platform, 43 at health centre, 46 at first level hospital, 22 at tertiary level, and 12 population-based interventions). The first stage thus established “unconstrained” priorities which, when costed, were well above the fiscal space for public health expenditure. Based on the government’s strategic decision to prioritise primary healthcare, a district package was designed covering three platforms (community, health centre, and first-level hospital). Out of the 151 interventions across the five platforms already prioritised, the result was a district level package covering only 117 interventions with an overall per-capita cost of US$ 29.7.

 A *second stage* of prioritisation was required to ensure that the cost of the district package did not exceed the fiscal space for health. Working closely with the World Bank, we estimated that the overall fiscal space for financing the public health sector would range from US$ 15 to US$ 21 per capita, depending on the extent of prioritisation of health within the total public expenditure. Taking the most optimistic of these scenarios and assuming an allocation of around 60% of expenditure at the district level, the MNHSR&C arrived at a limit of US$ 13 per capita for the package in its first year of implementation. The aim was to develop an affordable package that could be implemented immediately until health allocations increased to match the costs of the full EPHS. For this purpose, the NAC, during its second meeting held in June 2020, explored three options:

1. Further prioritise the interventions included in the package during the first stage and reduce them to the level that could be covered by the available public funding, using cost-effectiveness levels and the voting of the TWGs during deliberations on prioritisation of interventions.
2. Reduce the per capita cost by applying a 20% reduction in the cost of first-level hospital interventions as a co-payment.
3. Reduce the number of interventions to fit the available fiscal space by selecting interventions based on cost-effectiveness alone.

The NAC decided to recommend *Option 1* of a publicly financed, more limited immediate implementation package (IIP) of district-level interventions, covering the community, health centre, and first-level hospital platforms. Initially, 76 interventions were considered for the IIP. *Option 2* was not advised as any co-payment was regarded by the NAC as compromising the package’s goal of financial risk protection, especially as all interventions in *Option 1* were considered essential and of high priority to the country. *Option 3* was also not recommended because prioritisation would rely solely on cost-effectiveness, which was largely based on global evidence, and would ignore other important criteria, such as financial risk protection and equity.

 The IIP was then reviewed by the IAG in July 2020, which gave its recommendations to the NAC. These recommendations were subjected to a detailed review by the various departments and programmes of the MNHSR&C. The recommendations made by the IAG and the MNHSR&C resulted in a final IIP of 88 interventions at a cost of US$ 12.98. The final iterations of both IIP and full EPHS were presented to and approved in October 2020 by the UHC-EPHS SC and the Inter-ministerial Health and Population Council.

 Torres-Rueda et al^16^ discuss the inclusion and exclusion of interventions throughout the course of the deliberation process and the supplementary files of the paper contain detailed information on the status of interventions per stage in the deliberation process.

###  Final Package 

 We describe here the full district-level package as well as the IIP and their final costs and potential DALYs averted. Table S4 of Supplementary file 3 provides the full list of prioritised interventions, including target coverage and cost per capita at Year 2 of implementation.

**Figure 1:**
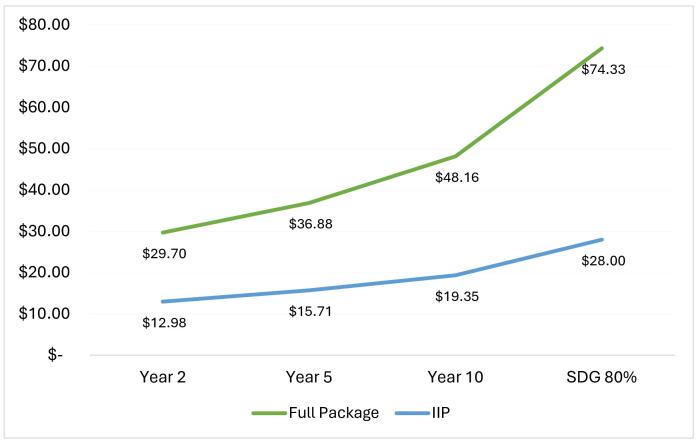
Estimated Cost of the Full and Immediate Implementation Packages Along the Universal Health Coverage Target of Achieving 80% Coverage. Abbreviations: SDG, Sustainable Development Goal; IIP, immediate implementation package.

Figure 1 shows the estimated total cost of both packages at initial implementation and at different points along the timeline for achieving the UHC target.

 The figure clearly shows that the full package cannot be implemented with the current level of public spending on health and that the IIP is the affordable package that can be implemented immediately until a more ambitious package can be considered if and when health allocations are significally increased in the future.

**Figure 2:**
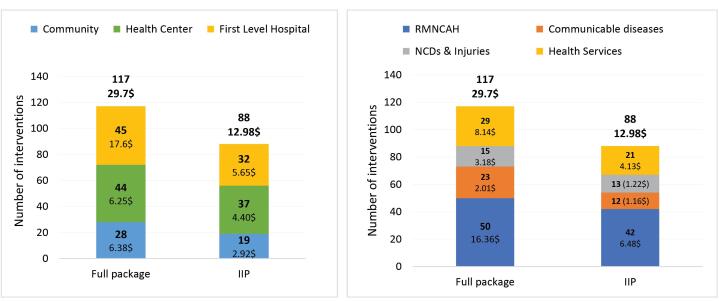
Cost Breakdown of Full Essential Package of Health Services and Immediate Implementation Package by Platform and Cluster. Abbreviations: RMNCAH, reproductive, maternal, neonatal, child and adolescent health; NCDs, non-communicable diseases; IIP, immediate implementation package.

Figure 2 presents the number and cost of interventions by platform and cluster in the full package and IIP.

 Most interventions in the full district EPHS (76%) are delivered at the health centre and first-level hospital platforms. A similar pattern is seen in the IIP. The largest number of interventions in the full package relate to RMNCAH (43% of the total number of interventions in the full package), followed by health services access (25%), communicable diseases (19%), and NCDs and injuries (13%). Most of the IIP interventions are at the RMNCAH cluster (47%), followed by health services access (24%), NCDs and injuries (15%), and communicable diseases (14%).

 The full package has an initial per capita cost of US$ 29.7, compared to US$ 12.98 for the IIP. Figure 2 shows that first-level hospital interventions in the full package are leading in terms of costs (39% of the total cost of the full package). Although the number of interventions is almost similar in the health centre platform, their cost is only about a third of the hospital interventions. The IIP has more health centre interventions than at the first-level hospital, but the collective cost is still higher in the hospital platform.

 Table provides comprehensive information on the contents of the full package and IIP by cluster and platform as well as on the impact of the two packages on disease burden. A review of the total DALYs averted shows that reducing the number of the interventions in the IIP resulted in only a modest reduction in DALYs averted, indicating that disease burden was a major criterion considered during the prioritisation process. Overall, implementing the full package would result in almost 47 million DALYs averted, around half of the estimated disease burden in Pakistan, while implementing the IIP would avert over 40 million DALYs, representing 43% of the burden of disease in the country.^30^ Furthermore, for both the full package and the IIP, the cost per DALY averted is lowest at the health centre level, which offers the largest number of EPHS interventions and is the backbone of primary healthcare in most countries. This platform also serves as a backup to the community level platform and is pivotal for referrals to the first level hospital. Strengthening health centre capacity is critical for the successful implementation of EPHS, although all interventions in the other delivery platforms are essential.

**Table:** Cost and DALYs Averted of Full Essential Package of Health Services and Immediate Implementation Package Interventions by Platform and Cluster

	**Cluster**	**Community**			**Health Centre**			**First Level Hospital**			**Total**		
		**Interventions**	**Cost Capita (US$)**	**DALYs Averted (Million)**	**Interventions**	**Cost Capita** **(US$)**	**DALYs Averted** **(Million)**	**Interventions**	**Cost Capita** **(US$)**	**DALYs Averted** **(Million)**	**Interventions**	**Cost Capita** **(US$)**	**DALYs Averted** **(Million)**
Full package	RMNCAH	17	5.42	7.16	15	2.17	19.22	18	8.77	2.40	50	16.36	28.78
	CDs	9	0.54	0.14	10	0.82	2.05	4	0.66	8.78	23	2.01	10.96
	NCDs	1	0.42	0.09	9	0.54	0.16	5	2.22	0.43	15	3.18	0.68
	Health services	1	0.002	0.0004	10	2.73	0.47	18	5.41	5.85	29	8.14	6.32
	**Subtotal**	**28**	**6.38**	**7.39**	**44**	**6.25**	**21.9**	**45**	**17.06**	**17.46**	**117**	**29.70**	**46.75**
IIP	RMNCAH	15	2.03	6.62	13	2.14	19.22	14	2.31	1.43	42	6.48	27.27
	CDs	3	0.47	0.05	7	0.59	1.95	2	0.11	8.57	12	1.16	10.57
	NCDs	1	0.42	0.09	9	0.54	0.16	3	0.26	0.36	13	1.22	0.61
	Health services	0	0	-	8	1.15	0.14	13	2.98	1.77	21	4.13	1.92
	**Subtotal**	**19**	**2.92**	**6.76**	**37**	**4.40**	**21.47**	**32**	**5.65**	**12.13**	**88**	**12.98**	**40.37**

Abbreviations: DALYs, disability-adjusted life years; IIP, immediate implementation package; CDs, communicable diseases; NCDs, non-communicable diseases; RMNCAH, reproductive, maternal, newborn, child, and adolescent health.

 In addition to the district-level package, 12 prioritised population-based interventions will be implemented, with an added cost of US$ 0.78 per capita (Supplementary file 3, Table S5). The implementation of these interventions is a joint mandate of the federal and provincial governments. Thus, the full district level package will, in effect, contain 129 interventions and the IIP will include 100 interventions, with an overall per capita cost of US$ 30.48 and US$ 13.76, respectively.

 The package could be implemented in a stepwise manner between the beginning of implementation in 2022 and 2030, as health budgets improve. Although the estimated cost of the IIP could be covered by existing public health expenditure during the first two years, the increasing population coverage levels to reach the UHC target will result in a concomitant rise in cost beyond what is currently available (Figure 1). Here again, the government will therefore have to explore potential approaches of increasing health allocations to expand the coverage of IIP between now and the time when 80% coverage is achieved for all interventions.

 Unlike the population-based interventions, which will have to be implemented along with the IIP, 22 tertiary care interventions (Supplementary file 4, Table S6) were prioritised and costed at US$ 6.5 per capita but were not recommended for immediate implementation. These were considered for inclusion in packages offered by the social health insurance schemes for tertiary care services, which most provinces are currently initiating.

###  Provincialisation and Piloting

**Figure 3:**
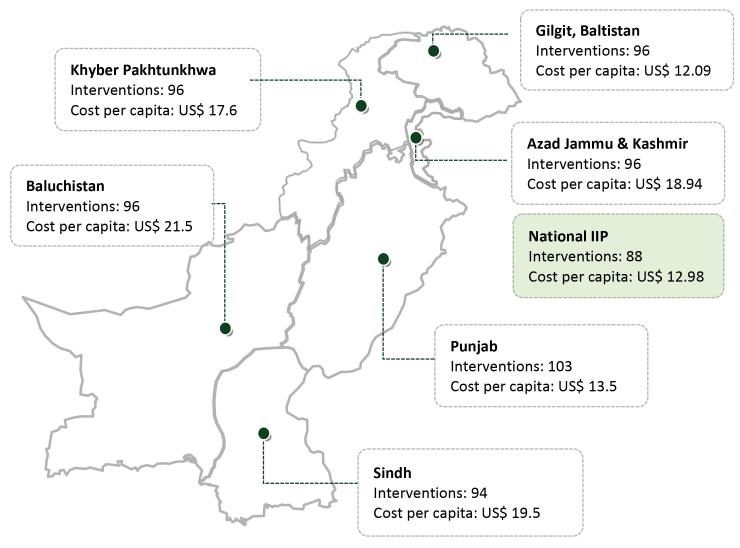
Adaptation of the National Essential Package of Health Services into Provincial Packages. Abbreviation: IIP, immediate implementation package.

 Pakistan is a devolved country and the substantial diversity between provinces necessitated a provincial adaptation of the national package prior to its implementation. Six district-level provincial packages were created through a process similar to the national EPHS design to better respond to local needs. The number of interventions and cost of packages varies across provinces (Figure 3). For example, the district package for the province of Sindh has 94 interventions, while the Punjab package has 103. Similarly, the implementation cost per capita ranges from US$ 12.9 in Gilgit, Baltistan to US$ 21.5 in Balochistan. While the provincial EPHS are largely similar in terms of interventions, some differences are due to the higher burden of specific health conditions, such as malaria in the provinces of Balochistan and Khyber Pakhtunkhwa. The difference in cost per capita is largely due to higher operational costs of implementing the package in some provinces. The provincial adaptation and costing were conducted largely by the local capacity built during the development of the national EPHS.

 A five-year investment case^31^ has been recommended to support an initial implementation in 12 districts across all provinces and federating areas during the first 30 months of Phase I of the implementation, followed by expansion to 40 districts under Phase II, covering a population of approximately 60 million. In addition to domestic funding, the implementation of the provincial EPHS is expected to be partly financed by a World Bank soft loan of US$ 300 million and with grant assistance from the Global Financing Facility, Bill & Melinda Gates Foundation, Global Alliance on Vaccines and Immunization, and the Global Fund under the National Health Support Programme.

## Discussion

 Over the last two decades, a considerable number of countries have developed basic packages of health services.^32-34^ However, the endorsement of UHC as a key SDG health target has reinvigorated an increasing number of countries,^18,35-39^ including Pakistan, to use the DCP3 approach and evidence in developing or updating their UHC packages of essential health services.

 National ownership and leadership of the process were key in developing the EPHS in Pakistan. Package development was at the centre of the national health vision and reforms, with specific commitment to initiate the process based on the DCP3 evidence. Ownership was demonstrated by a comprehensive governance structure, with the involvement of development partners at the outset.^13,18-19^

 The EPHS design was guided by the prioritisation approach, structure, and contents of the DCP3 EUHC package. By endorsing the DCP3 approach to advance UHC, the government has also committed to publicly finance the package and to adopt the progressive universalism approach.^40^

 Notwithstanding the difficulties brought on by the COVID-19 pandemic, which coincided with the process, the EPHS development faced multiple challenges that were inherent in Pakistan’s overall health system performance. There was a dearth of local data and evidence and low capacity to collect, verify, and analyse data.^20^ In addition, the evidence on cost-effectiveness of interventions could only partially be attributed to regionally generated studies. There were limitations to the relevance and applicability of evidence derived from global databases.^14^ Similar experience has been reported in Ethiopia with the use of standard tools to generate cost-effectiveness evidence and cost estimates.^41,42^ Despite these constraints, the experience from Ethiopia and Pakistan^14^ shows that judicious use of the available economic evaluation data contributes to prioritising high-impact interventions.

 The lack of institutional capacity, although a challenge in the initial stages of EPHS development, was effectively overcome by the support of established academic institutions and a committed team in the MNHSR&C and the DCP3 Secretariat capable of undertaking analysis and technical work. The challenge of having a wide range of stakeholders was addressed by establishing an elaborate governance structure and by adopting a participatory decision-making process.^13,19^

 A key component of package design is to critically assess the health system capacity and existing health financing mechanisms. Although such assessment was initiated following package design as part of the 2021-2026 Investment Case,^31^ it would have been more effective if health financing mechanisms and health system review had been systematically conducted early on as part of the preparatory assessment or at least concurrently with the prioritisation process. In addition, a major proportion of primary healthcare services are delivered by the private sector in Pakistan^23,43^ but their role and scope in implementing the EPHS has not received due consideration.

 Despite its initial success, the Pakistan experience provides important lessons learned. First, a robust process of societal dialogue and community engagement in decision-making was not conducted, which would have helped in determining how the public perceives their priority health needs and in gaining their support for the health reforms. In this context, Thailand and Tunisia have had rich experience of participatory governance as part of their health reform process.^44,45^

 Second, a stronger engagement of the planning and finance sectors, which control the public purse, would have resulted in a better understanding of current and future opportunities and the extent to which domestic financing could be made available to implement the package across the SDG timeline. Early engagement of the Ministry of Finance is also essential for a robust assessment of fiscal space and realistic planning for options of increased health allocation.

 Third, work on assessing the health system should be undertaken concurrently with package design to ensure a realistic and implementable package.

 Fourth, there is a strong need for institutionalisation of the process and for continued capacity building in Pakistan. The DCP3 Country Translation Project gave particular emphasis to building analytical capacity within the MNHSR&C in priority setting, economic evaluation, and setting and revising EPHS. Most of the capacity built was through learning-by-doing, supported in part by external experts. A positive spinoff was the successful adaptation of the national EPHS into provincial packages, which was primarily done by national staff trained during the development of the national EPHS. However, it is critical to retain the current human resources and skills within the MNHSR&C and partner institutions to achieve sustained institutionalisation of UHC-related processes.

 Finally, efforts to estimate the fiscal space for health should inevitably be tied to the macroeconomic analysis and assessment of the country’s prospects for economic growth. Considering the current economic forecast and the effects of the COVID-19 pandemic in Pakistan, it was not considered feasible to rely on economic growth to generate new resources. In such a situation, other options that demanded consideration were to: (*a*) enhance the efficient use of available resources at least partly by implementing an evidence-informed EPHS, (*b*) generate new health sector-specific resources through earmarked public health taxes on tobacco, unhealthy foods and beverages, (*c*) increase health allocation by reprioritising the government budget, (*d*) mobilise additional resources through external financing, and (*e*) build implementation and improvement capacity to deliver health services with greater efficiency.

 Additionally, the Pakistan experience provided some lessons learned for consideration in updating the DCP3 model packages. While the EUHC is a valuable tool and a good starting point to guide country work, there is a need for a better-defined and more specific definition of interventions. Some are too generic or have multiple components requiring several clinical actions. Although the scope of the proposed interventions covers a wide range of essential services needed in LLMICs, some critical interventions remain missing, notably in the areas of emergency medical services and pandemic preparedness and response. Furthermore, clinical guidelines are being concurrently developed, which can help in arriving at a proper diagnosis of the most common diseases with which the patients present and against which the essential interventions have been identified in the EPHS.

 The review of EPHS design in countries using the DCP3 evidence in recent years, including in Pakistan, has shown that the cost of the EUHC interventions is significantly higher than what Pakistan and many other LLMICs can realistically afford given the limited public health spending^18,20^; this is likely to be true even for the highest priority package of 108 interventions.

 In general, package development is a dynamic exercise that needs to be revisited at regular intervals to respond to policy changes and address evolving levels of disease burden and emerging health challenges.

## Conclusion

 Setting an evidence-informed EPHS that is fully endorsed by the government and the decision to move into implementation are promising achievements in Pakistan. However, this does not necessarily mean that implementation is readily feasible without addressing health system gaps. There are important lessons learned that Pakistan and other LLMICs should consider.

 Three elements are central to the development of an EPHS. First, it is critical to decide and act on how to finance the EPHS implementation within the timeframe of the SDG agenda, taking into account the increasing coverage levels to achieve UHC by 2030. Second, it is equally important to strengthen the health system to the level that allows for effective EPHS implementation, thereby establishing stronger public sector service delivery models while ensuring the active engagement of the private sector in providing primary care services. Third, the importance of institutionalisation and capacity building within ministries of health as a prerequisite for the sustenance of EPHS well beyond the life of timebound projects cannot be overemphasised.

## Ethical issues

 Ethical approvals were obtained from the London School of Hygiene and Tropical Medicine (21247) and Aga Khan University (2019-1992-5190); MoH clearance is being sought.

## Competing interests

 Authors declare that they have no competing interests.

## Funding

 This paper is part of a series of papers coordinated by the DCP3 Country Translation Project, which is funded by the Bill & Melinda Gates Foundation [Grant number: OPP1201812]. The sponsor had no involvement in paper design; collection, analysis and interpretation of the data; and in the writing of the paper.

## Supplementary files


General Appendix contains Figure S1, Box S1, And Tables S1 and S2.

Supplementary file 1 contains Table S3.

Supplementary file 2 contains Figures S2 and S3.

Supplementary file 3 contains Tables S4 and S5.

Supplementary file 4 contains Table S6.
