# Supplementary material for: Addressing the UHC Challenge Using the Disease Control Priorities 3 Approach: Lessons Learned and an Overview of the Pakistan Experience
Source: Int J Health Policy Manag. 2023 Dec 16;13:8003. doi: 10.34172/ijhpm.2023.8003 (PMC11607589; doi:10.34172/ijhpm.2023.8003)
Supplement: Supplementary file 1 — General Appendix contains Figure S1, Box S1, And Tables S1 and S2. [file ijhpm-13-8003-s001.pdf]

General Appendix contains Figure S1, Box S1, And Tables S1 and S2

Figure S1. *Governance Structure EPHS Pakistan*

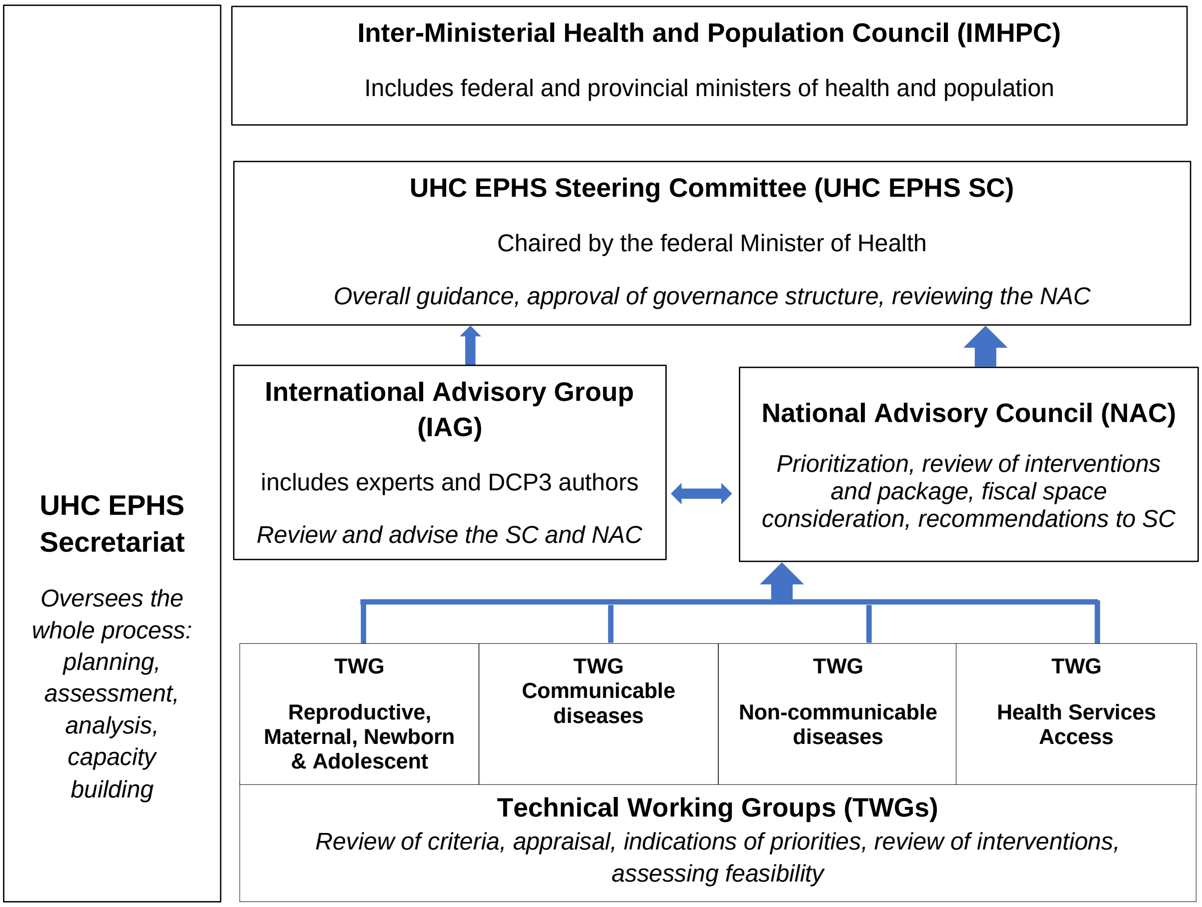

Note: Arrows represent the flow of information. Reporting obligations are represented by larger arrows

Box S1: *Further description on methods*

Study context and background

Initial steps

The Pakistan essential package of health services (EPHS) development process used Disease Control Priorities 3 (DCP3), which provides a global essential universal health coverage (EUHC) model package of 218 interventions, as a framework of reference.[1] In April 2019 the Ministry of National Health Services, Regulations & Coordination (MNHSR&C) of Pakistan carried out a scoping review and consultations with provincial-level stakeholders and the Health Planning, System Strengthening & Information Analysis Unit (HPSIU), to compare the composition of the 218 EUHC interventions to existing services and discuss their relevance to the Pakistani context. An initial shortlist of 170 EUHC interventions was suggested for further assessment.

EUHC interventions are delivered in five delivery platforms: community-level, health centre, first-level hospitals, referral hospitals, and population-level. During the process the MNHSR&C decided to focus on a district package of services. Consequently, nearly all population-level interventions were excluded, as they are operated and implemented at the national level (one was adapted for delivery at the community level in Pakistan). Of the remaining 159 interventions, nine were broken down into multiple interventions because either the scope of the EUHC interventions were deemed to be too broad to assess, or the intervention could be delivered in multiple platforms. Consequently, a final shortlist of 170 Pakistan-adapted interventions was recommended for formal assessment and appraisal.

Evidence and assessment of criteria

An evidence-informed deliberative process was used to prioritise these 170 interventions with the aim of defining an actionable, publicly funded package within fiscal space. Eight decision criteria for assessment were selected by the MNHSR&C: effectiveness, cost-effectiveness, budget impact, avoidable burden of disease, feasibility, equity, financial risk protection, and socio-economic impact.[2] The process of evaluating these criteria is detailed below.

Effectiveness: Effectiveness was selected but not considered explicitly as EUHC interventions are widely proven to be effective.

Cost-effectiveness: Data on incremental cost-effectiveness ratios (ICERs) were largely sourced from the Tufts Medical School Global Health Cost-Effectiveness Analysis registry and localised, covering 88 interventions. Remaining ICERs were sourced from HIP Tool default values[3], which in turn were sourced from DCP3. ICER values extracted and further details on methods used can be found elsewhere.[4] The ICERs of each individual intervention were ranked from high to low and categorised evenly: the bottom third were classified as having high cost-effectiveness, the middle third medium cost-effectiveness and the top third low cost-effectiveness). Their applicability to the Pakistani setting was rated on a scale of 1 (lowest applicability) to 3 (highest applicability).[4] Cost-effectiveness was also defined colloquially as 'health gained for money spent'.

Budget impact: The unit costs of DCP3 interventions considered were calculated through a normative, ingredients-based, bottom-up costing approach and used to determine budget impact. Further details are covered by Raza et al.[5] A mark-up factor of 1.6 was applied to unit costs to account for above-service delivery costs.[6] A total national cost per intervention per year was then calculated by multiplying the marked-up unit costs by the estimated population in need provided by the health management system and other health sector reporting systems. Total costs were then divided by the total population of Pakistan to calculate a cost per capita per intervention. This figure was then compared to health spending per capita and presented as a percentage of the health budget. As with ICERs, interventions were categorised evenly between those with high, medium and low budget impact.

Avoidable burden of disease: Pakistan-specific data, in the form of disability-adjusted life years (DALYs), were obtained from the Global Burden of Disease database from the Institute of Health Metrics and Evaluation.[7] The number of DALYs averted assigned to each intervention were obtained from the HIP Tool.[3]

Feasibility: The HPSIU prepared detailed descriptions of the resources used for each intervention. No scoring was done of feasibility. Full summary sheets are presented in the government report.[8]

Equity, financial risk protection and socio-economic impact: Criteria were not quantified as sufficient intervention-specific data were unavailable. However, during the appraisal process the committee used a definition of equity as prioritising interventions for vulnerable groups, and highlighted where interventions reached those groups, as part of their deliberations.

Evidence products

Three evidence products combining the abovementioned evidence, where available, were produced: (a) intervention descriptions sheets detailing intervention-specific resource use of high-quality service provision across a number of inputs,[8] (b) evidence sheets containing intervention-specific data on cost-effectiveness, budget impact, avoidable burden of disease,[2] and (c) presentations of package 'scenarios', which were produced using the HIPTool[3] and a Microsoft Excel-based model to allow for visual representation of multiple optimisation scenarios simultaneously.

Scenarios examined varied several parameters, including affordability, which explored two different fiscal space scenarios (US$ 8 and US$16), multiple payer options (assuming 20% patient co-payments for first-level hospital interventions), different target intervention coverage rates (at 80% and below) and a range of time horizons.

Appraisal of evidence process

Following an initial (i) shortlisting from the 218 DCP3 EUHC interventions, described above, evidence was reviewed and appraised by different stakeholders, in a sequential process. At each stage of the appraisal process, a recommendation on whether to prioritise or deprioritise an intervention was agreed upon and documented. Recommendations at each stage were non-binding; a recommendation to prioritise or deprioritise an intervention at one stage could be reversed at a subsequent stage.

For appraisal purposes, interventions were prioritised in two steps according to platform: community-based and HC interventions, and first-level hospital and referral hospital interventions. An initial (ii) technical working group (TWG) review of community-based and HC interventions (TWG1) was carried out in November 2019 by technical experts who prioritised interventions into three categories (high-priority, medium priority or not prioritised), followed by a (iii) National Advisory Council (NAC) meeting (NAC1), where stakeholders reviewed the recommendations from TWG and proposed a list of prioritised interventions in November 2019.

A (iv) second TWG reviewed evidence on first-line hospital and referral-hospital interventions (TWG2) in January 2020 following the same processes as TWG1. Then, a (v) second NAC (NAC2) meeting was convened in June 2020 with a broader remit: reviewing both the recommendations from NAC1 and the list of prioritised interventions from TWG2, hence covering interventions in all platforms.

Two key distinctions about NAC2 merit highlighting. Firstly, for the first time in the process, stakeholders had to simultaneously consider interventions across all platforms in the health system within a given fiscal space. As outlined above, several scenarios, including those which were budget constrained were introduced to crystalise trade-offs. Stakeholders were presented with scenarios prioritising all high priority interventions and high and medium priority interventions (as defined by the TWGs), as well as six implementation scenarios, highlighting different fiscal space constraints, time horizons, co-payments, and coverage rates. Further, a key decision around the objective of the

process, which emerged during NAC2, was to proceed with the design of two health benefit packages, reflecting different time horizons and fiscal space challenges: a reduced immediate implementation package (IIP) to be rolled out over 2 years, and the full EPHS, to be implemented in stepwise manner over the following decade as health budgets improve.[9] Following the NAC2, the IIP was reviewed by the International Advisory Group (IAG) in July 2020, which provided feedback. Lastly, the final iterations of both IIP and full EPHS were reviewed and approved by the UHC EPHS Steering Committee (UHC-EPHS SC) and the Inter-Ministerial Population Health Council (IMPHC) in October 2020.

Analysis of costs, outcomes and prioritised criteria during the process

The definition of a 'prioritised intervention' varies between appraisal stages and reflects the aim of each stage. Stakeholders in the initial shortlisting from DCP3 EUHC were asked to remove interventions not relevant to Pakistan and those in the TWGs were asked to group interventions by levels of priority. As the package was progressively evolving, the aim of these stages was not to propose a full implementable package (contrary to NAC2 and subsequent stages), nevertheless, we include all five stages in our analysis to analyse broad patterns in how different stakeholders prioritise and deprioritise interventions over time.

References

1. Watkins DA, Jamison DT, Mills A, et al. Universal Health Coverage and Essential Packages of Care. In: Jamison DT, Gelband H, Horton S, et al., eds. *Disease Control Priorities, Third Edition (Volume 9): Improving Health and Reducing Poverty*. 3rd ed. The International Bank for Reconstruction and Development / The World Bank; 2017. doi:10.1596/978-1-4648-0527-1_ch3
2. Baltussen R, Jansen M, Akhtar SS, et al. The use of evidence-informed deliberative processes for designing the universal health coverage benefit package in Pakistan. *Int J Heal Policy Manag*. 2023;(Under production).
3. University College of London (UCL). HIPTool Health Interventions Prioritization Tool 2020.
4. Huda M, Kitson N, Saadi N, et al. Assessing global evidence on cost-effectiveness to inform development of Pakistan's Essential Package of Health Services. *Int J Heal Policy Manag*. 2023;(Under production).
5. Raza W, Zulfiqar W, Shah MM, et al. Costing Interventions for Developing an Essential Package of Health Services: Application of a Rapid Method and Results from Pakistan. *Int J Health Plann Manage*. 2023;(Under production).
6. WHO Commission on Macroeconomics and Health. *Macroeconomics and Health : Investing in Health for Economic Development / Report of the Commission on Macroeconomics and Health*.; 2001.
7. Institute for Health Metrics and Evaluation (IHME). Global Burden of Disease Study data for Pakistan. 2019. https://ghdx.healthdata.org/gbd-2019.
8. Government of Pakistan, World Health Organization, Disease Control Priorities 3 (DCP3). Towards universal health coverage: Interventions' description of essential package of health services/ UHC benefit package of Pakistan. 2020. https://dcp-3.org/sites/default/files/resources/Interventions Description of Essential Package of Health Services UHC Benefit Package of Pakistan 2020.pdf?issu.
9. Alwan A, Siddiqi S, Malik S, et al. Addressing the UHC challenge using the Disease Control Priorities 3 approach: experience from Pakistan and lessons learned. *Int J Heal Policy Manag*. 2023;(Under production)

Table S1: Evidence Products. Types of evidence included in the assessment and stage of the appraisal process used

Abbreviations: TWG1: Technical Working Group 1. TWG2: Technical Working Group 2. NAC1: National Advisory Council 1. NAC2: National Advisory Council 2.

		**EVIDENCE SHEETS**	**INTERVENTION DESCRIPTION SHEETS**	**OPTMISATION MODELS**
	**Decision criterion 1: Effectiveness**	Not assessed: interventions from DCP3 EUHC package considered to be effective.	Not assessed: interventions from DCP3 EUHC package considered to be effective.	Not assessed: interventions from DCP3 EUHC package considered to be effective.
**EVIDENCE TYPE COLLECTED OR COLLATED**	**Decision criterion 2: Cost-effectiveness**	ICERs were ranked and categorised into low, medium and high cost-effectiveness, or no cost-effectiveness evidence. ICER applicability to Pakistan was assessed on a scale of 1 (lowest) to 3 (highest).	Intervention descriptions sheets were used to describe service delivery and to consequently compare with interventions found in the global cost-effectiveness literature in order to select relevant ICERs.	Selected ICERs were used in the HIP Tool-based and Excel-based optimisation models.
	**Decision criterion 3: Budget impact**	Costs per capita were presented as an absolute figure and percentage of total health spending per capita. Budget impact was categorised as low, medium and high.	Intervention descriptions sheets were used to understand resource use and consequently to calculate unit costs per intervention.	Unit costs were used in HIP Tool-based optimisation models. Costs per capita were used in Excel-based optimisation models.
	**Decision criterion 4: Avoidable burden of disease**	Avoidable burden of disease was presented was categorised as low, medium and high.	Not used	DALYs averted per intervention were included in the HIPTool-based and Excel-based optimisation models.
	**Decision criterion 5: Feasibility**	No data was collated/collected but the criterion was listed in evidence sheet to elicit expert opinions during deliberations.	Resource use was described across a number of inputs (staff level and time, medicines, diagnostics, supplies and equipment).	Not used
	**Decision criterion 6: Equity**	No data was collated/collected but the criterion was listed in evidence sheet to elicit expert opinions during deliberations.	Not used	Not used
	**Decision criterion 7: Financial risk protection**	No data was collated/collected but the criterion was listed in evidence sheet to elicit expert	Not used	Not used

		opinions during deliberations.		
	**Decision criterion 8: Socio-economic impact**	No data was collated/collected but the criterion was listed in evidence sheet to elicit expert opinions during deliberations.	Not used	Not used
	**Quality of cost-effectiveness**	ICERs were categorised as having low, medium or high applicability to the Pakistani setting.	Not used	Not used
	**Fiscal space**	No data was collated/collected but the criterion was listed in evidence sheet to elicit expert opinions during deliberations.	Not used	Assumptions included in the Excel-based optimisation models.
	**Co-payments**	Not used	Not used	Assumptions included in the Excel-based optimisation models.
	**Time horizon**	Not used	Not used	Assumptions included in the HIP Tool-based and Excel-based optimisation models.
	**Coverage**	Not used	Not used	Data on current and future coverage rates per intervention were used in the HIP Tool-based and Excel-based optimisation models.
**APPRAISAL PROCESS STAGE WHERE EVIDENCE PRODUCT WAS USED**		TWG1, NAC1, TWG2, NAC2, Final packages	TWG1, NAC1, TWG2, NAC2, Final packages	NAC1, NAC2, Final packages

Table S2: Definition of categorisation of interventions

Criteria	Methods for categorisation
(1) Cost-effectiveness	Interventions were categorised as having high, medium or low cost-effectiveness (or having no available data) based on their classification in the evidence sheets. ICERs were ranked in numerical order and divided evenly into three groups before each technical working group (TWG). Interventions in the group with lowest ICERs were classified as having high-cost effectiveness, and those in the group with the highest ICERs were classified as having low cost-effectiveness.
(2) Budget impact	Interventions were categorised as having high, medium or low budget impact (or no available data) based on their classification in the evidence sheets: low budget impact= total cost is <0.5% of total budget, medium budget impact is 0.5%-1% of total budget, high budget impact is >1% of total budget.
(3) Burden of preventable disease	Interventions were categorised as preventing a high, medium or low burden of disease (or no data available) based on their classification in the evidence sheets. DALYs averted per intervention were obtained from the HIP Tool and the IHME. Total DALYs averted were ranked in numerical order and divided evenly into three groups before each TWG. The interventions in the group with the lowest number of total DALYs averted were classified as preventing low burden of disease and those in the group with the highest number of total DALYs averted were classified as preventing a high burden of disease.
(4) ICER quality	ICERs were categorised as having low, medium or high applicability to the Pakistani setting. See Huda et al. for further details.[1]
(5) Current coverage	Current coverage data were procured by the Health Planning, System Strengthening & Information Analysis Unit at the Ministry of National Health Services, Regulations & Coordination of Pakistan. Specific coverage rates are presented in Appendix 6. Categories were constructed: no current coverage= 0%, low current coverage 1%-20%, medium current coverage= 21%-40%, high current coverage= 41%-100%.
(5) Delivery platform	Delivery platforms were categorised as per DCP3 Essential Universal Health Coverage (EUHC) package interventions[2]: community-based, primary health care, first-level hospitals and referral hospitals.
(6) Intervention cluster	Intervention clusters were categorised as per DCP3 EUHC package interventions: Reproductive, maternal, neonatal and child health, infectious diseases, non-communicable diseases and injury prevention and care, and health services.[2]
(7) Intervention purpose	Interventions were divided by their primary purpose using World Health Organization Universal Health Coverage categories: promotive, preventative, curative, rehabilitative and palliative.[3]
(8) Vulnerable population	Interventions addressing the needs of vulnerable populations where those involving reproductive, maternal, neonatal and child health (as per NAC guidance).
(9) Rule of rescue	Interventions categorised as not involving or involving the rule of rescue, which was defined as the imperative to rescue identifiable individuals facing avoidable death.[4]

Source:

(1) Huda M, Kitson N, Saadi N, et al. Assessing global evidence on cost-effectiveness to inform development of Pakistan's Essential Package of Health Services. *Int J Heal Policy Manag*. 2023;(Under Submission).

(2) Watkins DA, Jamison DT, Mills A, et al. Universal Health Coverage and Essential Packages of Care. In: Jamison DT, Gelband H, Horton S, et al., eds. *Disease Control Priorities, Third Edition (Volume 9): Improving Health and Reducing Poverty*. 3rd ed. The International Bank for Reconstruction and Development / The World Bank; 2017. doi:10.1596/978-1-4648-0527-1_ch3

(3) World Health Organization. Universal health coverage (UHC). 2021. Accessed November 21, 2021. https://www.who.int/news-room/fact-sheets/detail/universal-health-coverage-(uhc).

(4) McKie J, Richardson J. The rule of rescue. *Soc Sci Med*. 2003;56(12):2407-2419. doi: 10.1016/s0277-9536(02)00244-7
