# Supplementary material for: Addressing the UHC Challenge Using the Disease Control Priorities 3 Approach: Lessons Learned and an Overview of the Pakistan Experience
Source: Int J Health Policy Manag. 2023 Dec 16;13:8003. doi: 10.34172/ijhpm.2023.8003 (PMC11607589; doi:10.34172/ijhpm.2023.8003)
Supplement: Supplementary file 2 — Supplementary file 1 contains Table S3. [file ijhpm-13-8003-s002.pdf]

Article title: Addressing the UHC Challenge Using the Disease Control Priorities 3 Approach: Lessons Learned and an Overview of the Pakistan Experience

Journal name: International Journal of Health Policy and Management (IJHPM)

Authors' information: Ala Alwan[1]*, Sameen Siddiqi[2], Malik Safi[3], Raza Zaidi[3], Muhammad Khalid[3], Rob Baltussen[4], Ina Gudumac[1], Maryam Huda[2], Maarten Jansen[4], Wajeeha Raza[5], Sergio Torres-Rueda[6], Wahaj Zulfiqar[3], Anna Vassall[6]

[1]DCP3 Country Translation Project, London School of Hygiene and Tropical Medicine, London, UK.

[2]Department of Community Health Sciences, Aga Khan University, Karachi, Pakistan.

[3]Ministry of National Health Services, Regulations and Coordination, Islamabad, Pakistan.

[4]Department of Health Evidence, Radboud Institute of Health Sciences, Radboud University Medical Center, Nijmegen, The Netherlands.

[5]Centre for Health Economics, University of York, York, UK.

[6]Department of Global Health & Development, London School of Hygiene and Tropical Medicine, London, UK.

***Correspondence to:** Ala Alwan; Email: aalwan1@outlook.com

Citation: Alwan A, Siddiqi S, Safi M, et al. Addressing the UHC challenge using the Disease Control Priorities 3 approach: lessons learned and an overview of the Pakistan experience. Int J Health Policy Manag. 2023;12:8003. doi:10.34172/ijhpm.2023.8003

Supplementary file 1

Table S3. Mapping of available DCP3 EUHC interventions by cluster

Cluster[(a)]	EUHC interventions	# Available interventions in Pakistan	General availability	Limited availability
RMNCAH	67	50	22 (44%)	28 (56%)
Communicable diseases	52	32	10 (31%)	22 (69%)
NCDs & Injuries	45	16	6 (37.5%)	10 (62.5%)
Health Services Access	54	37	4 (11%)	33 (89%)
**Total**	**218**	**135**	**42 (31%)**	**93 (69%)**

[(a)] Clusters matching definitions from DCP3

Abbreviations: RMNCAH: reproductive, maternal, newborn, child, and adolescent health; NCDs: non-communicable diseases
