# Supplementary material for: Addressing the UHC Challenge Using the Disease Control Priorities 3 Approach: Lessons Learned and an Overview of the Pakistan Experience
Source: Int J Health Policy Manag. 2023 Dec 16;13:8003. doi: 10.34172/ijhpm.2023.8003 (PMC11607589; doi:10.34172/ijhpm.2023.8003)
Supplement: Supplementary file 3 — Supplementary file 2 contains Figures S2 and S3. [file ijhpm-13-8003-s003.pdf]

Article title: Addressing the UHC Challenge Using the Disease Control Priorities 3 Approach: Lessons Learned and an Overview of the Pakistan Experience

Journal name: International Journal of Health Policy and Management (IJHPM)

Authors' information: Ala Alwan[1]*, Sameen Siddiqi[2], Malik Safi[3], Raza Zaidi[3], Muhammad Khalid[3], Rob Baltussen[4],Ina Gudumac[1], Maryam Huda[2], Maarten Jansen[4], Wajeeha Raza[5], Sergio Torres-Rueda[6], Wahaj Zulfiqar[3], Anna Vassall[6]

[1]DCP3 Country Translation Project, London School of Hygiene and Tropical Medicine, London, UK.

[2]Department of Community Health Sciences, Aga Khan University, Karachi, Pakistan.

[3]Ministry of National Health Services, Regulations and Coordination, Islamabad, Pakistan.

[4]Department of Health Evidence, Radboud Institute of Health Sciences, Radboud University Medical Center, Nijmegen, The Netherlands.

[5]Centre for Health Economics, University of York, York, UK.

[6]Department of Global Health & Development, London School of Hygiene and Tropical Medicine, London, UK.

*__Correspondence to:__ Ala Alwan; Email: aalwan1@outlook.com

Citation: Alwan A, Siddiqi S, Safi M, et al. Addressing the UHC challenge using the Disease Control Priorities 3 approach: lessons learned and an overview of the Pakistan experience. Int J Health Policy Manag. 2023;12:8003. doi:10.34172/ijhpm.2023.8003

Supplementary file 2

Figure S2. Evidence Summary Sheet

Evidence summary

Intervention HC38: Provision of aspirin for all cases of suspected acute myocardial infarction

Cost-effectiveness	Cost-effectiveness rank order	Quality of ICER
Medium: 443	31/86	☆☆☆

Burden of Disease
High: 2,514

Budget impact	Cost per capita	Secondary/ tertiary care budget impact
Low	0.66 PKR	No

Also consider:

☐ Feasibility ☐ Financial risk protection

☐ Equity ☐ Social and economic impact

Figure S3. Example of a description sheet

C. Non-Communicable Diseases and Injury Prevention Cluster

C.11. Cardiovascular, Respiratory and Related Disorders Package of Services

C.11.120. Provision of aspirin for all cases of suspected acute myocardial infarction

DCP3 code: HC38

Platform:

PHC Level

- 8-6 BHU/ 24-7 BHU/RHC/ Health Centre/ GP Clinic

Process:

Patient registration at reception (Receptionist)

Consultation (Doctor/ Nurse/ LHV)

Recommended Method

- Immediate management: Sublingual aspirin

Referral to higher facility for treatment

Follow up

Medicines:

Aspirin: 75 mg

HMIS Tools:

1. Recording Tool: OPD Ticket, OPD register
2. Reporting Tool: Monthly report
3. Client/Patient Card: Follow up visit card
4. IEC material: Leaflet, Flip chart

Supervision:

- EDO Health, Deputy DHO, THO

Standard Protocol:

- Aspirin Therapy
- Role of primary health care in prevention and control of CVDs

National Training Curriculum/ Guidelines:

- Not Available

Reference Material:

- Prevention of Cardiovascular Disease Guidelines for Assessment and Management of Cardiovascular Risk WHO 2007
- Global Atlas on Cardiovascular Disease Prevention and Control WHO

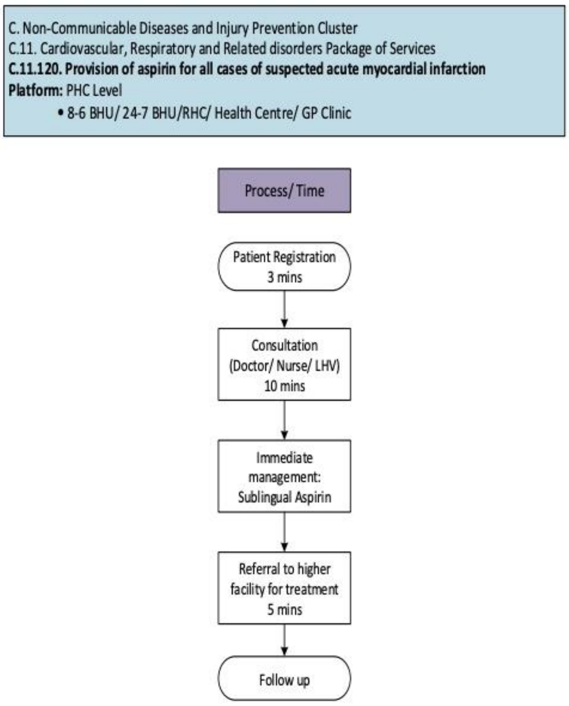

Example of a description sheet developed for each EPHS intervention. The full list is available at: Ministry of National Health Services, Regulations, and Coordination, Disease Control Priorities 3 (DCP3), World Health Organization. Interventions' description of Essential Package of Health Services/ UHC Benefit Package of Pakistan. Islamabad: MoNHSR&C; 2020. http://dcp-3.org/resources/interventions-description-essential-package-health-services-uhc-benefit-package-pakistan-0
