# Supplementary material for: Addressing the UHC Challenge Using the Disease Control Priorities 3 Approach: Lessons Learned and an Overview of the Pakistan Experience
Source: Int J Health Policy Manag. 2023 Dec 16;13:8003. doi: 10.34172/ijhpm.2023.8003 (PMC11607589; doi:10.34172/ijhpm.2023.8003)
Supplement: Supplementary file 4 — Supplementary file 3 contains Tables S4 and S5. [file ijhpm-13-8003-s004.pdf]

Article title: Addressing the UHC Challenge Using the Disease Control Priorities 3 Approach: Lessons Learned and an Overview of the Pakistan Experience

Journal name: International Journal of Health Policy and Management (IJHPM)

Authors' information: Ala Alwan[1]*, Sameen Siddiqi[2], Malik Safi[3], Raza Zaidi[3], Muhammad Khalid[3], Rob Baltussen[4],Ina Gudumac[1], Maryam Huda[2], Maarten Jansen[4], Wajeeha Raza[5], Sergio Torres-Rueda[6], Wahaj Zulfiqar[3], Anna Vassall[6]

[1]DCP3 Country Translation Project, London School of Hygiene and Tropical Medicine, London, UK.

[2]Department of Community Health Sciences, Aga Khan University, Karachi, Pakistan.

[3]Ministry of National Health Services, Regulations and Coordination, Islamabad, Pakistan.

[4]Department of Health Evidence, Radboud Institute of Health Sciences, Radboud University Medical Center, Nijmegen, The Netherlands.

[5]Centre for Health Economics, University of York, York, UK.

[6]Department of Global Health & Development, London School of Hygiene and Tropical Medicine, London, UK.

*__Correspondence to:__ Ala Alwan; Email: aalwan1@outlook.com

Citation: Alwan A, Siddiqi S, Safi M, et al. Addressing the UHC challenge using the Disease Control Priorities 3 approach: lessons learned and an overview of the Pakistan experience. Int J Health Policy Manag. 2023;12:8003. doi:10.34172/ijhpm.2023.8003

Supplementary file 3

Table S4 – List of Pakistan District EPHS Interventions

Legend

✓	**Full District EPHS**
✓	**Immediate Implementation District EPHS**
✓	**Special Initiative**

#	Code/ Platform	Full Name of Intervention	Cluster	Full District EPHS	IIP	Special Initiatives	Coverage Year 2	Spending Year 2	Cost per Capita Year 2
1	C1-COM	Antenatal and postpartum education on family planning	RMNCH	✓	✓		39%	1,671,965	0.01
2	C2-COM	Counselling of mothers on providing thermal care for preterm new-borns (delayed bath and skin-to-skin contact)	RMNCH	✓	✓		20%	400,301	0
3	C3a-COM	Management of labour and delivery in low-risk women by skilled attendant	RMNCH	✓	✓		20%	7,630,418	0.03
4	C3b-COM	Basic neonatal resuscitation following delivery	RMNCH	✓	✓		20%	534,342	0
5	C3c-PHC	Management of labour and delivery in low-risk women by skilled attendant	RMNCH	✓	✓		70%	64,378,904	0.29
6	C3d-PHC	Basic neonatal resuscitation following delivery	RMNCH	✓	✓		70%	4,712,215	0.02
7	C4-COM	Promotion of breastfeeding or complementary feeding by lay health workers	RMNCH	✓	✓		39%	2,867,185	0.01

8	C5-PHC	Tetanus toxoid immunization among schoolchildren and among women attending antenatal care	RMNCH	✓	✓		64%	47,757,266	0.21
9	C8-COM	Detection and management of acute severe malnutrition and referral in the presence of complications	RMNCH	✓		✓	52%	327,870,307	1.47
10	C10-COM	Education on handwashing and safe disposal of children's stools	RMNCH	✓	✓		39%	10,352,967	0.05
11	C11-COM	Pneumococcus vaccination	RMNCH	✓	✓		78%	86,123,282	0.39
12	C12-COM	Rotavirus vaccination	RMNCH	✓	✓		72%	39,267,286	0.18
13	C14-COM	Provision of vitamin A and zinc supplementation to children according to WHO guidelines, and provision of food supplementation to women and children in food insecure households	RMNCH	✓		✓	60%	428,302,759	1.92
14	C16-COM	Childhood vaccination series (diphtheria, pertussis, tetanus, polio, BCG, measles, hepatitis B, Hib, rubella)	RMNCH	✓	✓		70%	78,688,206	0.35
15	C18-COM	Education of schoolchildren on oral health	RMNCH	✓	✓		39%	19,434,644	0.09
16	C19-COM	Vision pre-screening by teachers; vision tests and provision of ready-made glasses on-site by eye specialists	RMNCH	✓	✓		39%	42,725,968	0.19
17	C27a-COM	Provision of iron and folic acid supplementation to pregnant women, and provision of food or caloric supplementation to pregnant women in food insecure households (CL)	RMNCH	✓	✓		60%	158,674,132	0.71
18	C27b-PHC	Provision of iron and folic acid supplementation to pregnant women, and provision of food or caloric supplementation to pregnant women in food insecure households (PHC)	RMNCH	✓	✓		60%	106,639,304	0.48

19	C28-COM	Community-based HIV testing and counselling (for example, mobile units and venue-based testing), with appropriate referral or linkage to care and immediate initiation of lifelong ART	Infectious Disease	✓		✓	12%	337,032	0
20	C30a-COM	Provision of condoms to key populations, including sex workers, men who have sex with men, people who inject drugs (IDU), transgender populations, and prisoners	Infectious Disease	✓		✓	20%	5,681,703	0.03
21	C30b-COM	Provision of disposable syringes to people who inject drugs (IDU)	Infectious Disease	✓		✓	20%	545,269	0
22	C32-COM	Routine contact tracing to identify individuals exposed to TB and link them to care	Infectious Disease	✓		✓	39%	7,918,664	0.04
23	C33-PHC	For malaria due to P. vivax, test for G6PD deficiency; if normal, add chloroquine or chloroquine plus 14-day course of primaquine	Infectious Disease	✓			44%	5,329,603	0.02
24	C43-COM	Early detection and treatment of leishmaniasis, dengue, chikungunya, rabies, trachoma, and helminthiasis	Infectious Disease	✓	✓		40%	14,944,171	0.07
25	C45-COM	Identify and refer patients with high risk including pregnant women, young children, and those with underlying medical conditions	Infectious Disease	✓			50%	1,326,180	0.01
26	C46-COM	In the context of an emerging infectious outbreak, provide advice and guidance on how to recognize early symptoms and signs and when to seek medical attention	Infectious Disease	✓	✓		50%	20,620,041	0.09
27	C51-COM	WASH behaviour change interventions, such as community-led total sanitation	NCD & IPC	✓	✓		39%	94,164,478	0.42
28	C53a-COM	Identification/screening of early childhood development indicators, including motor, sensory and language stimulation	Health Services	✓			2%	447,502	0.002
29	C53b-PHC	Early childhood development rehabilitation interventions, including motor, sensory, and language	Health Services	✓			15%	36,434,357	0.16

		stimulation							
30	HC1-PHC	Early detection and treatment of neonatal pneumonia with oral antibiotics	RMNCH	✓	✓		55%	4,273,684	0.02
31	HC2-PHC	Management of miscarriage or incomplete abortion and post abortion care	RMNCH	✓	✓		30%	10,138,544	0.05
32	HC3-FLH	Management of preterm premature rupture of membranes, including administration of antibiotics	RMNCH	✓	✓		30%	30,897,363	0.14
33	HC4a-COM	Provision of condoms and hormonal contraceptives, including emergency contraceptives	RMNCH	✓	✓		14%	4,099,860	0.02
34	HC4b-PHC	Provision of condoms and hormonal contraceptives, including emergency contraceptives and IUDs	RMNCH	✓	✓		14%	6,149,790	0.03
35	HC5a-COM	Counselling of mothers on providing kangaroo care for new-borns (CL)	RMNCH	✓	✓		2%	29,620	0
36	HC5b-PHC	Counselling of mothers on providing kangaroo care for new-borns (PHC)	RMNCH	✓	✓		2%	44,430	0
37	HC6-FLH	Management of neonatal sepsis, pneumonia, and meningitis using injectable and oral antibiotics	RMNCH	✓	✓		30%	6,943,795	0.03
38	HC7-PHC	Pharmacological termination of pregnancy	RMNCH	✓	✓		22%	3,089,340	0.01
39	HC9a-COM	Screening of hypertensive disorders in pregnancy	RMNCH	✓	✓		54%	719,518	0
40	HC9b-PHC	Screening and management of hypertensive disorders in pregnancy	RMNCH	✓	✓		54%	17,915,764	0.08
41	HC10-FLH	Screening and management of diabetes in pregnancy (gestational diabetes or pre-existing type II diabetes)	RMNCH	✓	✓		10%	992,290	0
42	HC11-PHC	Management of labour and delivery in low-risk women (BEmNOC), including initial treatment of	RMNCH	✓	✓		70%	147,617,840	0.66

		obstetric or delivery complications prior to transfer							
43	HC12-PHC	Detection and treatment of childhood infections with danger signs (IMCI)	RMNCH	✓	✓		22%	5,675,811	0.03
44	HC14-PHC	Psychological treatment for mood, anxiety, ADHD, and disruptive behaviour disorders	RMNCH	✓			5%	2,468,190	0.01
45	HC16-PHC	Post gender-based violence care, including counselling, provision of emergency contraception, and rape-response referral (medical and judicial) - to be executed as inter-sectoral intervention	RMNCH	✓			5%	4,544,273	0.02
46	HC17-PHC	Syndromic management of common sexual and reproductive tract infections (for example urethral discharge, genital ulcer, and others) according to WHO guidelines	RMNCH	✓	✓		52%	57,797,840	0.26
47	HC19-FLH	For individuals testing positive for hepatitis B and C, assessment of treatment eligibility by trained providers followed by initiation and monitoring of antiviral treatment when indicated	Infectious Disease	✓		✓	12%	121,669,698	0.55
48	HC20-PHC	Hepatitis B and C testing of individuals identified in the national testing policy (based on endemicity and risk level), with appropriate referral of positive individuals to trained providers	Infectious Disease	✓		✓	24%	15,583,330	0.07
49	HC21-PHC	Partner notification and expedited treatment for common STIs, including HIV	Infectious Disease	✓	✓		42%	33,863,370	0.15
50	HC23-PHC	Provider-initiated testing and counselling for HIV, STIs, and hepatitis, for all in contact with health system in high-prevalence settings, including prenatal care with appropriate referral or linkage to care including immediate ART initiation for those testing positives for HIV	Infectious Disease	✓		✓	25%	30,795,092	0.14

51	HC24-FLH	As resources permit, hepatitis B vaccination of high-risk populations, including healthcare workers, PWID, MSM, household contacts, and persons with multiple sex partners	Infectious Disease	✓			15%	585,521	0
52	HC25-PHC	Provision of male circumcision service	Infectious Disease	✓	✓		24%	28,938,406	0.13
53	HC26-PHC	For PLHIV and children under five who are close contacts or household members of individuals with active TB, perform symptom screening and chest radiograph; if there is no active TB, provide isoniazid preventive therapy according to current WHO guidelines	Infectious Disease	✓	✓		2%	2,404,714	0.01
54	HC27-PHC	Diagnosis of TB, including assessment of rifampicin resistance using rapid molecular diagnostics (Ultra expert), and initiation of first-line treatment per current WHO guidelines for drug-susceptible TB; referral for confirmation, further assessment of drug resistance, and treatment of drug-resistant TB	Infectious Disease	✓	✓		70%	38,658,963	0.17
55	HC28-COM	Screening for HIV in all individuals with a diagnosis of active TB; if HIV infection is present, start (or refer for) ARV treatment and HIV care	Infectious Disease	✓		✓	14%	204,654	0
56	HC30-PHC	Evaluation and management of fever in clinically stable individuals using WHO IMAI guidelines, with referral of unstable individuals to first-level hospital care	Infectious Disease	✓	✓		55%	12,405,679	0.06
57	HC32-PHC	Provision of insecticide-treated nets to children and pregnant women attending Health Centre	Infectious Disease	✓	✓		33%	10,311,022	0.05
58	HC33-PHC	Identify and refer to higher levels of health care patients with signs of progressive illness	Infectious Disease	✓	✓		30%	4,319,630	0.02
59	HC36-PHC	Long-term combination therapy for persons with multiple CVD risk factors, including screening for CVD in community settings using non-lab-based tools	NCD & IPC	✓	✓		7%	5,887,615	0.03

		to assess overall CVD risk							
60	HC37-PHC	Low-dose inhaled corticosteroids and bronchodilators for asthma and for selected patients with COPD	NCD & IPC	✓	✓		24%	21,005,045	0.09
61	HC38-PHC	Provision of aspirin for all cases of suspected acute myocardial infarction	NCD & IPC	✓	✓		22%	477,948	0
62	HC39a-PHC	Screening of albuminuria kidney disease including targeted screening among people with diabetes	NCD & IPC	✓	✓		10%	11,246,414	0.05
63	HC41-PHC	Secondary prophylaxis with penicillin for rheumatic fever or established rheumatic heart disease	NCD & IPC	✓	✓		20%	840,387	0
64	HC42-PHC	Treatment of acute pharyngitis in children to prevent rheumatic fever	NCD & IPC	✓	✓		25%	1,602,323	0.01
65	HC45-PHC	Opportunistic screening for hypertension for all adults and initiation of treatment among individuals with severe hypertension and/or multiple risk factors	NCD & IPC	✓	✓		22%	40,740,352	0.18
66	HC50-PHC	Management of depression and anxiety disorders with psychological and generic antidepressant therapy	NCD & IPC	✓	✓		10%	36,974,544	0.17
67	HC56-PHC	Targeted screening for congenital hearing loss in high-risk children using otoacoustic emissions testing	NCD & IPC	✓	✓		1%	906,113	0
68	HC57a-PHC	Dental extraction (PHC)	Health Services	✓	✓		40%	92,790,726	0.42
69	HC57b-FLH	Dental extraction (FLH)	Health Services	✓	✓		45%	80,079,483	0.36
70	HC58a-PHC	Drainage of dental abscess (PHC)	Health Services	✓	✓		30%	34,877,338	0.16
71	HC59-PHC	Drainage of superficial abscess	Health Services	✓	✓		35%	34,388,923	0.15

72	HC60-PHC	Management of non-displaced fractures	Health Services	✓	✓		30%	5,791,518	0.03
73	HC61-PHC	Resuscitation with basic life support measures	Health Services	✓	✓		30%	141,643	0
74	HC62-PHC	Suturing laceration	Health Services	✓	✓		40%	4,871,371	0.02
75	HC63a-PHC	Treatment of caries	Health Services	✓	✓		20%	60,788,300	0.27
76	HC64-PHC	Basic management of musculoskeletal and neurological injuries and disorders, such as prescription of simple exercises and sling or cast provision	Health Services	✓	✓		30%	21,724,528	0.1
77	HC68-PHC	Health centre pathology services<br>(Note: Cost included in relevant interventions)	Health Services	✓			8%	316,032,586	1.42
78	FLH1-FLH	Detection and management of foetal growth restriction	RMNCH	✓			45%	450,318,604	2.02
79	FLH3-FLH	Jaundice management with phototherapy	RMNCH	✓	✓		42%	4,458,454	0.02
80	FLH4-FLH	Management of eclampsia with magnesium sulphate, including initial stabilization at Health Centre	RMNCH	✓	✓		50%	40,770,626	0.18
81	FLH5-FLH	Management of maternal sepsis, including early detection at Health Centre	RMNCH	✓	✓		45%	46,067,773	0.21
82	FLH6-FLH	Management of new-born complications infections, meningitis, septicaemia, pneumonia and other very serious infections requiring continuous supportive care (such as IV fluids and oxygen)	RMNCH	✓	✓		44%	19,735,564	0.09
83	FLH7-FLH	Management of preterm labour with corticosteroids, including early detection	RMNCH	✓			45%	384,256,163	1.72

84	FLH8-FLH	Management of labour and delivery in high-risk women, including operative delivery (CEmNOC)	RMNCH	✓	✓		45%	257,561,989	1.16
85	FLH10-FLH	Surgical termination of pregnancy by manual vacuum aspiration and dilation and curettage	RMNCH	✓	✓		25%	2,421,900	0.01
86	FLH11-FLH	Full supportive care for severe childhood infections with danger signs	RMNCH	✓			45%	412,527,270	1.85
87	FLH12-FLH	Management of severe acute malnutrition associated with serious infection	RMNCH	✓			37%	194,164,486	0.87
88	FLH13-FLH	Early detection and treatment of early-stage cervical cancer	RMNCH	✓	✓		2%	86,019	0
89	FLH14-FLH	Insertion and removal of long-lasting contraceptives (IUCDs and Implants)	RMNCH	✓	✓		4%	503,257	0
90	FLH15-FLH	Tubal ligation	RMNCH	✓	✓		12%	71,274,663	0.32
91	FLH16-FLH	Vasectomy	RMNCH	✓	✓		1%	659,929	0
92	FLH17-FLH	Referral of cases of treatment failure for drug susceptibility testing; enrolment of those with MDR-TB for treatment per WHO guidelines (either short or long regimen)	Infectious Disease	✓	✓		63%	11,750,124	0.05
93	FLH18-FLH	Evaluation and management of fever in clinically unstable individuals using WHO IMAI guidelines, including empiric parenteral antimicrobials and antimalarials and resuscitative measures for septic shock	Infectious Disease	✓	✓		45%	12,193,860	0.05
94	FLH20-FLH	Management of acute coronary syndromes with aspirin, unfractionated heparin, and generic thrombolytics (when indicated)	NCD & IPC	✓			30%	314,595,982	1.41
95	FLH22-FLH	Management of acute exacerbations of asthma and COPD using systemic steroids, inhaled beta-agonists,	NCD & IPC	✓		✓	22%	123,546,297	0.55

		and, if indicated, oral antibiotics and oxygen therapy							
96	FLH23-FLH	Medical management of acute heart failure	NCD & IPC	✓	✓		33%	41,163,969	0.18
97	FLH24-FLH	Management of bowel obstruction	NCD & IPC	✓	✓		45%	15,570,284	0.07
98	FLH30-FLH	Management of intoxication/poisoning syndromes using widely available agents; e.g., activated charcoal, naloxone, bicarbonate, antivenin	NCD & IPC	✓	✓		20%	403,151	0
99	FLH31-FLH	Appendectomy	Health Services	✓	✓		50%	147,959,396	0.66
100	FLH34-FLH	Colostomy	Health Services	✓	✓		30%	2,840,128	0.01
101	FLH35-FLH	Escharotomy or fasciotomy	Health Services	✓	✓		12%	11,540,565	0.05
102	FLH36-FLH	Fracture reduction and placement of external fixator and use of traction for fractures	Health Services	✓	✓		45%	64,354,438	0.29
103	FLH38-FLH	Hysterectomy for uterine rupture or intractable postpartum haemorrhage	Health Services	✓	✓		60%	4,203,149	0.02
104	FLH39-FLH	Irrigation and debridement of open fractures	Health Services	✓	✓		40%	43,348,095	0.19
105	FLH41a-FLH	Management of septic arthritis	Health Services	✓	✓		20%	1,076,500	0
106	FLH41b-FLH	Placement of external fixation and use of traction for fractures	Health Services	✓	✓		25%	22,573,467	0.1
107	FLH42-FLH	Relief of urinary obstruction by catheterization or suprapubic cystostomy	Health Services	✓	✓		60%	246,929,810	1.11

108	FLH43-FLH	Removal of gallbladder including emergency surgery	Health Services	✓	✓		45%	29,636,400	0.13
109	FLH44-FLH	Repair of perforations (for example, perforated peptic ulcer, typhoid ileal perforation)	Health Services	✓	✓		65%	8,079,525	0.04
110	FLH45-FLH	Resuscitation with advanced life support measures, including surgical airway	Health Services	✓			20%	35,768,895	0.16
111	FLH48a-FLH	Trauma laparotomy	Health Services	✓		✓	62%	62,940,922	0.28
112	FLH49-FLH	Trauma-related amputations	Health Services	✓		✓	26%	23,132,326	0.1
113	FLH50-FLH	Tube thoracostomy	Health Services	✓			25%	6,082,301	0.03
114	FLH52-FLH	Compression therapy for amputations, burns, and vascular or lymphatic disorders	Health Services	✓	✓		40%	2,163,866	0.01
115	RH1-FLH	Full supportive care for preterm new-borns	RMNCH	✓	✓		25%	32,688,553	0.15
116	RH14-FLH	Cataract extraction and insertion of intraocular lens	Health Services	✓			65%	414,807,744	1.86
117	P5-COM	Systematic identification of individuals with TB symptoms among high-risk groups and linkage to care ("active case finding")	Infectious Disease	✓	✓		39%	68,222,543	0.31

Table S5 – List of 12 Prioritised Interventions at the Population Level

#	Code	Intervention	Cluster	Cost Per Capita (US$)
1	P1	Mass media messages concerning sexual and reproductive health and mental health for adolescents (also included in HIV and Mental health packages of services)	RMNCH	0.02
2	P2	Mass media messages concerning healthy eating or physical activity (also included in CVD and Musculoskeletal packages of services)	RMNCH	0.02
3	C25	Education campaign for the prevention of gender-based violence	RMNCH	0.02
4	P4	Mass media encouraging use of condoms, voluntary medical male circumcision and STI testing	RMNCH	0.02
5	P6	Sustained integrated vector management for effective control of visceral Leishmaniasis, dengue, chikungunya, CCHF, and other nationally important causes of non-malarial fever vector borne NTDs	Infectious Diseases	0.02
6	P13	Mass media messages concerning awareness on handwashing and health effects of household air pollution	Infectious Diseases	0.02
7	P7	Conduct a comprehensive assessment of International Health Regulations (IHR) competencies using the Joint External Evaluation (JEE) tool	Infectious Diseases	0.00
8	P10	Develop and implement a plan to ensure surge capacity in hospital beds, stockpiles of disinfectants, equipment for supportive care and personal protective equipment	Infectious Diseases	0.49
9	P11	Develop plans and legal authority for curtaining interactions between infected persons and un-infected population and implement and evaluate infection control measures in health facilities	Infectious Diseases	0.01
10	P8	Conduct simulation exercises and health worker training for outbreak events including outbreak investigation, contact tracing and emergency response	Infectious Diseases	0.00
11	P9	Decentralize stocks of antiviral medications to reach at risk groups and disadvantaged populations	Infectious Diseases	0.14
12	P3	Mass media messages concerning use of tobacco (also included in CVD package of services)	NCD & IPC	0.02
