# Supplementary material for: Addressing the UHC Challenge Using the Disease Control Priorities 3 Approach: Lessons Learned and an Overview of the Pakistan Experience
Source: Int J Health Policy Manag. 2023 Dec 16;13:8003. doi: 10.34172/ijhpm.2023.8003 (PMC11607589; doi:10.34172/ijhpm.2023.8003)
Supplement: Supplementary file 5 — Supplementary file 4 contains Table S6. [file ijhpm-13-8003-s005.pdf]

Article title: Addressing the UHC Challenge Using the Disease Control Priorities 3 Approach: Lessons Learned and an Overview of the Pakistan Experience

Journal name: International Journal of Health Policy and Management (IJHPM)

Authors' information: Ala Alwan[1]*, Sameen Siddiqi[2], Malik Safi[3], Raza Zaidi[3], Muhammad Khalid[3], Rob Baltussen[4],Ina Gudumac[1], Maryam Huda[2], Maarten Jansen[4], Wajeeha Raza[5], Sergio Torres-Rueda[6], Wahaj Zulfiqar[3], Anna Vassall[6]

[1]DCP3 Country Translation Project, London School of Hygiene and Tropical Medicine, London, UK.

[2]Department of Community Health Sciences, Aga Khan University, Karachi, Pakistan.

[3]Ministry of National Health Services, Regulations and Coordination, Islamabad, Pakistan.

[4]Department of Health Evidence, Radboud Institute of Health Sciences, Radboud University Medical Center, Nijmegen, The Netherlands.

[5]Centre for Health Economics, University of York, York, UK.

[6]Department of Global Health & Development, London School of Hygiene and Tropical Medicine, London, UK.

***Correspondence to:** Ala Alwan; Email: aalwan1@outlook.com

Citation: Alwan A, Siddiqi S, Safi M, et al. Addressing the UHC challenge using the Disease Control Priorities 3 approach: lessons learned and an overview of the Pakistan experience. Int J Health Policy Manag. 2023;12:8003. doi:10.34172/ijhpm.2023.8003

Supplementary file 4

Table S6 – List of prioritised interventions at tertiary level hospitals

#	Code	Title of Intervention	Cluster	Cost per capita (US$)
1	FLH25	Calcium and vitamin D supplementation for secondary prevention of osteoporosis	NCD & IPC	2.52
2	FLH33	Craniotomy for trauma	Health Services	0.00
3	FLH37b	Hernia Repair, including emergency surgery for neonates and infants	Health Services	0.00
4	FLH40	Management of osteomyelitis, including surgical debridement for refractory cases	Health Services	0.55
5	FLH41c	Management of septic arthritis	Health Services	0.00
6	FLH48b	Trauma laparotomy in children	Health Services	0.00
7	RH2	Specialized TB services, including management of MDR- and XDR-TB treatment failure and surgery for TB	Infectious Disease	0.06
8	RH3	Management of refractory febrile illness including etiologic diagnosis at reference microbiological laboratory	Infectious Disease	0.71
9	RH4	Management of acute ventilatory failure due to acute exacerbations of asthma and COPD; in COPD use of bilevel positive airway pressure preferred	NCD & IPC	0.00
10	RH5	Retinopathy screening via telemedicine, followed by treatment using laser photocoagulation	NCD & IPC	0.00
11	RH6	Use of percutaneous coronary intervention for acute myocardial infarction where resources permit	NCD & IPC	0.76
12	RH7	Treatment of early-stage breast cancer with appropriate multimodal approaches (including generic chemotherapy), with curative intent, for cases that are detected by clinical examination at Health Centre	NCD & IPC	0.66
13	RH8	Treatment of early-stage colorectal cancer with appropriate multimodal approaches (including generic chemotherapy), with curative intent, for cases that are detected by clinical examination at Health Centre	NCD & IPC	0.02
14	RH9	Treatment of early-stage childhood cancers (such as Burkitt and Hodgkin lymphoma, acute lymphoblastic leukaemia, retinoblastoma, and Wilms tumour) with curative intent in paediatric cancer units or hospitals	NCD & IPC	0.04
15	RH10	Elective surgical repair of common orthopaedic injuries (for example, meniscal and ligamentous tears) in individuals with severe functional limitation	NCD & IPC	0.22
16	RH11	Urgent, definitive surgical management of orthopaedic injuries (for example, by open reduction and internal fixation)	NCD & IPC	0.10
17	RH12	Repair of cleft lip and cleft palate	NCD & IPC	0.46
18	RH13	Repair of club foot	NCD & IPC	0.02
19	RH15	Repair of anorectal malformations and Hirschsprung's Disease	Health Services	0.02
20	RH16	Repair of obstetric fistula	Health Services	0.02
21	RH17	Insertion of shunt for hydrocephalus	Health Services	0.00
22	RH18	Surgery for trachomatous trichiasis	Health Services	0.07
